# Supplementary material for: Content-rich biological network constructed by mining PubMed abstracts
Source: BMC Bioinformatics. 2004 Oct 8;5:147. doi: 10.1186/1471-2105-5-147 (PMC528731; doi:10.1186/1471-2105-5-147)
Supplement: Additional File 2 — The original results of the above study (non-essential files are deleted to keep the file size under the limit set by BMC bioinformatics). [file 1471-2105-5-147-S2.bz2 › chilibotAdditionalFile2/dip20/473ID10725697E10558/html/GPR2_CCL27.html]

 


 **GPR2** and **CCL27** 
  
Found 11 abstracts in PubMed, retrieved 11.  
 

 What does Google say? 
 PDF only 
| .edu only 

---

**Interactive relationship** (e.g. stimulation, inhibition, etc)

**Neutral relationship**- The recently discovered CC chemokine ligand 27  [ **CCL27** ]   **CCL27** , through interaction with its cognate receptor, CC chemokine receptor 10 CCR10  [ **GPR2** ] , appears to be involved in attracting a subset of memory T cells to the skin during some cutaneous inflammatory responses.  Ref: 12718728 Expert Opin Biol Ther< MedlineTA>Expert Opin Biol Ther,
- These results provide evidence that  **GPR2**  is a specific receptor for ESkine  [ **CCL27** ] .  Ref: 10725696 J Immunol, 2000
- In vivo, intracutaneous  **CCL27**  injection attracted lymphocytes and, conversely, neutralization of  **CCL27**  CCR10  [ **GPR2** ]  interactions impaired lymphocyte recruitment to the skin leading to the suppression of allergen induced skin inflammation.  Ref: 11821900 Nat Med, 2002
- Together, these findings indicate that  **CCL27**  CCR10  [ **GPR2** ]  interactions have a pivotal role in T cell mediated skin inflammation.  Ref: 11821900 Nat Med, 2002
- **CCL27**  CCR10  [ **GPR2** ]  interactions regulate T cell mediated skin inflammation.  Ref: 11821900 Nat Med, 2002
- Cutting edge the orphan chemokine receptor G protein coupled receptor 2  [ **GPR2** ]  GPR 2  [ **GPR2** ] , CCR10  [ **GPR2** ]  binds the skin associated chemokine  **CCL27**  CTACK  [ **CCL27** ]  ALP  [ **CCL27** ]  ILC  [ **CCL27** ] .  Ref: 10725697 J Immunol, 2000
- Surprisingly, secreted  **CCL27**  can also reach the nucleus after CCR10  [ **GPR2** ]  mediated internalisation, where it may also be capable of directly modulating transcription to alter cell behaviour.  Ref: 12718728 Expert Opin Biol Ther< MedlineTA>Expert Opin Biol Ther, 2000
- In contrast,  **CCL27** , which is most similar to CCL28 and shares CCR10  [ **GPR2** ] , showed no such potent antimicrobial activity.  Ref: 12538707 J Immunol, 2003
- We report that  **CCL27**  binds the previously orphan chemokine receptor GPR 2  [ **GPR2** ] , as detected by calcium flux and chemotactic responses of GPR 2  [ **GPR2** ]  transfectants.  Ref: 10725697 J Immunol, 2000

**Non-interactive relationship** (e.g. studied together, co-existance, homology, etc.)

- CC chemokine receptor CCR 4 and the CCR10  [ **GPR2** ]  ligand cutaneous T cell attracting chemokine  [ **CCL27** ]  CTACK  [ **CCL27** ]  in lymphocyte trafficking to inflamed skin.  Ref: 11714760 J Exp Med, 2001
- The mAb 1B5 recognizes functional CCR10  [ **GPR2** ]  expression, as chemotactic responsiveness to cutaneous T cell attracting chemokine  [ **CCL27** ]  CTACK  [ **CCL27** ]   **CCL27**  a CCR10  [ **GPR2** ]  ligand parallels the staining of TH subsets.  Ref: 12406880 Blood, 2003
- Cutting edge identification of the orphan receptor G protein coupled receptor 2  [ **GPR2** ]  as CCR10  [ **GPR2** ] , a specific receptor for the chemokine ESkine  [ **CCL27** ] .  Ref: 10725696 J Immunol, 2000
- Because of the skin associated expression pattern of  **CCL27** , we focused on the expression of  **CCL27**  and CCR10  [ **GPR2** ]  in normal skin compared with inflammatory and autoimmune skin diseases.  Ref: 10725697 J Immunol, 2000
- Here we report the cloning of full length human h  **GPR2**  and mouse m  **GPR2**  cDNAs, and the identification of  **GPR2**  as a receptor for a novel CC chemokine called ESkine  [ **CCL27** ] .  Ref: 10725696 J Immunol, 2000
- CCL28 desensitized the calcium mobilization induced in CCR10  [ **GPR2** ]  transfectants by  **CCL27** , indicating that these chemokines share this new chemokine receptor.  Ref: 10781587 J Biol Chem, 2000
- **CCL27** , and MEC supports chemotaxis of transfected lymphoid cells expressing CCR10  [ **GPR2** ] , a known CTACK  [ **CCL27** ]  receptor.  Ref: 10975800 J Immunol, 2000
- As the CCR10  [ **GPR2** ]  ligand ESkine  [ **CCL27** ]   **CCL27**  had the highest identity to MC148 and because both chemokines are expressed in the skin we suspected MC148 to block CCR10  [ **GPR2** ] .  Ref: 11298347 Eur J Immunol, 2001
- The skin associated chemokine  **CCL27**  also called CTACK  [ **CCL27** ] , ALP  [ **CCL27** ]  and ESkine  [ **CCL27** ]  and its receptor CCR10  [ **GPR2** ]  GPR 2  [ **GPR2** ]  mediate chemotactic responses of skin homing T cells in vitro.  Ref: 11821900 Nat Med, 2002
